# Celiac Disease and Inflammatory Cardiomyopathies: Exploring the Heart-Gut Axis

**DOI:** 10.3390/jcm13226936

**Published:** 2024-11-18

**Authors:** Lucia Ilaria Birtolo, Gianluca Di Pietro, Riccardo Improta, Paolo Severino, Endrit Shahini, Carmine Dario Vizza

**Affiliations:** 1 Department of Clinical, Internal, Anesthesiological and Cardiovascular Sciences, Sapienza University of Rome, “Policlinico Umberto I” Hospital, Viale del Policlinico, 155, 00161 Rome, Italy; gianluca.dipietro@uniroma1.it (G.D.P.); riccardo.improta@uniroma1.it (R.I.); paolo.severino@uniroma1.it (P.S.); dario.vizza@uniroma1.it (C.D.V.); 2 Gastroenterology Unit, National Institute of Gastroenterology-IRCCS “Saverio de Bellis”, 70013 Castellana Grotte, Italy; endrit.shahini00@gmail.com

**Keywords:** celiac disease, cardiovascular events, pericarditis, myocarditis, inflammatory heart disease

## Abstract

**Background**: Celiac disease (CD) is associated with an increased risk of cardiovascular (CV) events, especially inflammatory heart diseases. We aimed to gather evidence on the association between CD and inflammatory CV diseases, including pericarditis and myocarditis, and the underlying pathophysiological mechanisms. **Methods**: The PubMed, Scopus, and Google Scholar databases were searched for studies assessing the prevalence and the long-term outcomes of patients with CD and inflammatory CV diseases. **Result**: Ten studies (nine case series/reports and one prospective observational study) covering a total of 22 patients, including 9 women (41%) and 13 men (59%), were evaluated. The median age was 23 (IQR, 12–41) years. A total of 6 patients had recurrent pericarditis, while 16 patients had celiac-related myocarditis (11 patients presented with heart failure and 5 with arrhythmia). A strict gluten-free diet (GFD) alone was effective in 87.5% of cases (4/6 for pericarditis and 10/16 for myocarditis). All patients with pericarditis achieved complete resolution of symptoms. Conversely, heart function was restored in 81% of patients with myocarditis. Only one patient died. **Conclusions**: Myocarditis and/or pericarditis, especially if recurrent, may complicate CD in pediatric and adult subjects due to an autoimmune reaction involving the pericardium/myocardium and the small intestine.

## 1. Introduction

Inflammatory cardiomyopathies, such as pericarditis and myocarditis, may be associated with systemic autoimmune diseases [1,2]. Previous studies have already highlighted a strong link between autoimmune disorders and accelerated coronary atherogenesis [3]. Recognizing and treating these autoimmune disorders is crucial as, if left untreated, they may impede recovery or even worsen myocardial function [3].

Celiac disease (CD) is a multisystemic autoimmune disorder characterized by chronic and permanent intolerance to gluten in genetically susceptible individuals. It results in inflammation of the small intestinal mucosa, villous atrophy, and crypt hyperplasia. The clinical expression of CD is highly variable, ranging from classic and non-classic gastrointestinal manifestations to extraintestinal manifestations and subclinical cases. It affects an average of 1% of the general population, and its prevalence is underestimated due to a lack of diagnosis, making it a global health concern [4].

CD can be associated with several autoimmune diseases, including autoimmune thyroid disease, IgA selective deficiency, dermatitis herpetiformis, osteoporosis, infertility, type 1 diabetes mellitus, primary biliary cirrhosis, primary sclerosing cholangitis, epilepsy with cerebral calcifications, and Down syndrome. Previous studies have shown that several cardiovascular (CV) conditions, including inflammatory cardiomyopathies, are more common in subjects with CD than in those without the disease [2]. However, the association between these CV conditions and CD has rarely been reported in the literature, highlighting the need for further research.

This updated review examines the latest evidence on the association between CD and inflammatory CV diseases in pediatric and adult subjects, including pericarditis and myocarditis, and the underlying pathophysiological mechanisms.

## 2. Methods

The current analysis adheres to the guidelines outlined in the Preferred Reporting Items for Systematic Reviews and Meta-analyses (PRISMA) [5]. Furthermore, it has been prospectively registered in the International Prospective Register of Systematic Reviews (PROSPERO CRD 42024582483). Ethical approval was not deemed necessary for this study-level review. This study’s publication did not involve individual patient information; hence, written consent from patients was not obtained.

### Search Strategy and Data Extraction

**Figure 1:**
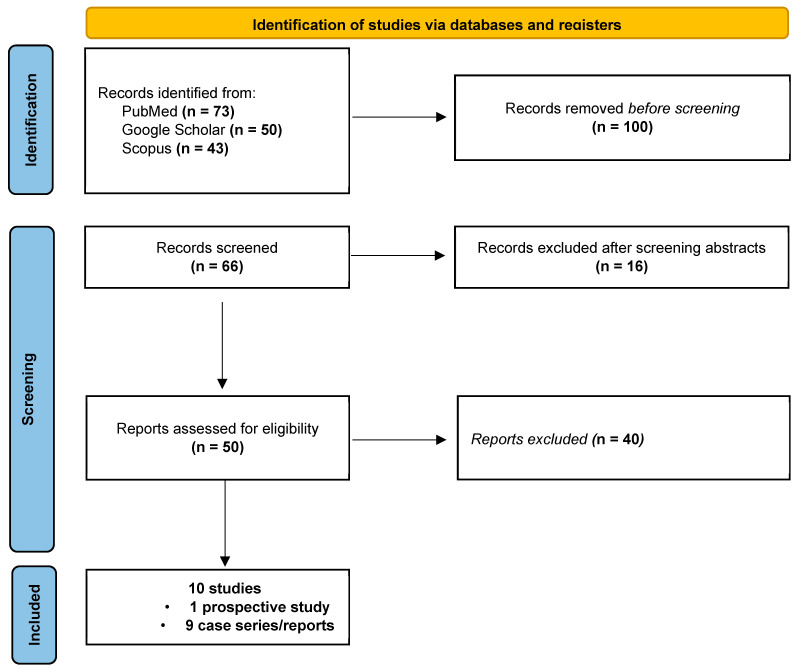
PRISMA 2020 flow diagram of the searching strategy.

We conducted a comprehensive search across electronic databases, including MEDLINE, Google Scholar, and Scopus, to identify all relevant studies discussing the long-term outcomes of inflammatory CV diseases in CD patients up to May 2024. Our search utilized a combination of keywords and MeSH terms, such as “(((((((((((((((celiac disease) OR (celiac sprue)) OR (nontropical sprue)) OR (gluten-sensitive enteropathy)) OR (enteropathy)) AND (pericarditis)) OR (acute pericarditis)) OR (pericardial effusion)) OR (pericarditis covid)) OR (recurrent pericarditis)) OR (constrictive pericarditis)) OR (noninfectious pericarditis)) OR (idiopathic pericarditis)) OR (infective pericarditis)) AND (myocarditis)) OR (inflammatory cardiomyopathy)”. Reference lists of identified articles were manually searched to uncover any other relevant studies. Two independent physicians (L.I.B, G.D.P.) screened the literature, excluding duplicated results, with disagreements resolved by a third author (E.S.). Figure 1 shows the PRISMA flow diagram illustrating the study selection process. Two authors (L.I.B., G.D.P.) extracted data independently, and a third author (E.S.) verified the results. The information collected included details on the investigators, the year of publication, the journal, the study design, the study period, the follow-up duration, the sample size, patient characteristics, and outcomes. Case reports are inherently biased. However, standardized tools have been developed for the assessment of the methodological quality of systematic reviews. For this reason, two authors (L.I.B., E.S.) independently evaluated the quality of the studies using a standardized tool adapted from Murad et al. [6] Lastly, the ROBIN I tool was used to estimate the bias of observational studies [7].

## 3. Results

We selected 10 studies (9 case series/reports and 1 prospective observational study) covering 22 patients. Among these patients, 9 were women (41%), whereas 13 were men (59%). The characteristics of the included studies are shown in Table 1. In brief, the median age was 23 (IQR 12–41) years. Six patients had recurrent pericarditis with chest rubs and electrocardiographic abnormalities as a consequence of CD.

**Figure 2:**
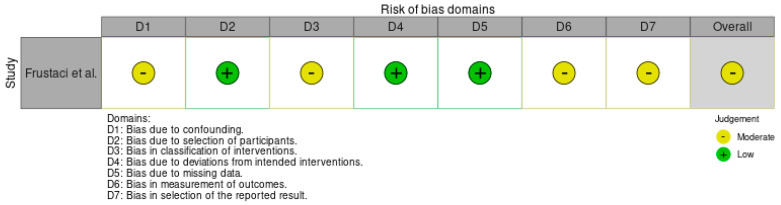
Risk bias assessment for observational studies (ROBIN I Tool) [7].

In contrast, myocarditis related to CD was observed in 16 patients with variable clinical presentation: 11 patients were admitted for heart failure and 5 for arrhythmia. Other associated symptoms included diarrhea, abdominal pain, iron deficiency anemia refractory to an oral iron replacement, and weight loss. Jejunal biopsy was used to diagnose CD and showed, in the majority of cases, villous atrophy, while antibodies were mainly used to monitor compliance with the gluten-free diet (GFD). Clinical evaluation supported by electrocardiograms, echocardiograms, and blood samples was required to diagnose myocardial and pericardial involvement. Cardiac magnetic resonance (CMR) was performed in only two patients in these studies. A strict GFD alone was effective in many cases of recurrent pericarditis (4/6 patients with pericarditis, 66.7%) and myocarditis (10/16 patients with myocarditis, 62.5%). Immunosuppressive therapy was required in a small percentage of cases (2/6 for pericarditis, 33.3%; 6/16 for myocarditis, 37.5%). Despite a GFD with additional immunosuppressive therapy, 3 out of 16 patients (18.75%) with myocarditis did not recover their heart function, and only 1 patient died. The quality assessment of case reports/series using the tool suggested by Murad et al. is shown in Table 2. Figure 2 reports the visual plot for risk bias assessment for observational studies using the ROBIN I tool (2016).

## 4. Discussion

### 4.1. Celiac Disease

The CD is a chronic autoimmune enteropathy caused by a unique genetic background [15]. This genetic predisposition is determined by the presence of either the HLA-DQ2 or HLA-DQ8 genes, or both, which serve as the “conditio sine qua non” for disease development. These genes play a crucial role in the onset of the disease process by facilitating the presentation of gluten-derived peptides to CD4+ T cells, which then initiate an immune-mediated response. The enzyme tissue transglutaminase (TG2) deamidates specific gluten peptides, increasing their affinity to HLA-DQ2 or HLA-DQ8. This results in a CD4+ T-helper 1 T-cell activation, which can activate cellular and humoral immune mechanisms, leading to damage to the small intestinal villi and the subsequent malabsorption of nutrients. The destruction of the villi results in a decreased surface area for absorption, leading to a reduced ability to absorb essential nutrients and water. The immune response also stimulates the production of serum antibodies, such as anti-tissue transglutaminase antibodies, which play a crucial role in diagnosing CD. Therefore, gluten sensitivity and the resulting immune response may account for the systemic reactions observed in CD.

The clinical presentation of CD is a complex spectrum, ranging from asymptomatic to symptomatic individuals. The symptoms can manifest in various ways, including abdominal pain, bloating, diarrhea, steatorrhea, weight loss, fatigue, iron deficiency, and anemia. Individuals may also present with extraintestinal symptoms, such as skin rashes (i.e., dermatitis herpetiformis), osteoporosis, infertility, and neurological symptoms [16]. The diagnosis of CD requires a comprehensive approach that involves a combination of serological and histological evaluations. Specifically, serological tests, including anti-tissue transglutaminase antibodies, are used for screening and diagnosing CD. The gold standard in diagnosing CD is the presence of histological changes in small-bowel mucosal biopsies, such as villous atrophy, crypt hyperplasia, and profound inflammation, which are the typical histological hallmarks of CD. The clinical presentation, genetics, and family history are likewise of utmost diagnostic relevance [17].

### 4.2. Celiac Disease and Inflammatory Cardiomyopathies

Inflammatory cardiomyopathies in CD patients have previously been reported with an estimated prevalence of 0.3% to 5%, significantly higher than in the general population [18]. However, the pathogenesis remains controversial, and several key mechanisms have been proposed. Antigenic mimicry, whereby the immune system mistakenly targets both intestinal and cardiac tissues due to structural similarities in their antigens, is the most prominent [18,19]. In addition, intestinal dysbiosis has recently been observed in CD patients, contributing to increased intestinal permeability and facilitating bacterial translocation [20,21]. The resulting chronic inflammatory state creates favorable conditions for promoting systemic inflammatory responses that can affect the myocardium [20,21]. In addition, patients with CD often suffer from malabsorption, resulting in deficiencies of essential nutrients (thiamine, selenium, calcium, and carnitine), which play a critical role in cardiac physiology [18,19]. Chronic anemia, which is common in untreated CD due to the poor absorption of iron and folic acid, increases cardiovascular stress by creating a hyperdynamic cardiovascular state that can lead to heart failure over time [18,19].

### 4.3. Pericarditis and Myocarditis

Myocarditis and pericarditis are inflammatory heart disorders with a general prevalence of approximately 10–20 events per 100,000 person-years [11].

They may present with variable clinical signs, symptoms, etiologies, and outcomes, including acute heart failure, sudden death, arrhythmia, and chronic dilated cardiomyopathy [22].

The most common causes of myocarditis and pericarditis are viral. However, non-infectious, drug/vaccine-associated hypersensitivity and autoimmune causes are less well defined [23]. A detailed and accurate history of investigating specific etiologies or causative agents such as drugs, systemic autoimmune disorders, virus infections, or infections caused by other infective agents is necessary to guide the diagnostic–0therapeutic process. These different causes result in potentially different inflammatory mechanisms and treatment responses.

The potential severity of myocarditis and pericarditis underscores the importance of prompt diagnosis and analysis of the underlying causes. These are crucial for immediate treatment and significantly promote the recovery of any already present myocardial dysfunction. Moreover, they can prevent the development of myocardial dysfunction and the potential worsening of chronic heart failure.

Regarding the diagnosis, according to international guidelines, acute myocarditis is diagnosed if at least one of the following elements are present in addition to a suggestive clinical presentation: (a) endomyocardial biopsy (EMB) suggestive of acute myocarditis and/or (b) CMR suggestive of acute myocarditis [23,24,25,26].

Similarly, acute pericarditis is diagnosed if at least two of the four criteria below are met: (a) pericardial chest pain; (b) pericardial rubs; (c) electrocardiogram findings such as PR depression and new widespread ST elevation; or (d) imaging evidence of pericardial effusion (new or worsening). Elevated inflammatory markers (i.e., C-reactive protein, erythrocyte sedimentation rate, leucocytosis) or evidence of pericardial inflammation by imaging techniques such as CMR or computed tomography (CT) further support the diagnosis [27].

### 4.4. Celiac Disease and Pericarditis

The majority of cases of pericarditis are idiopathic [28,29]. A viral etiology is often implicated but rarely proven [29]. In some cases of pericarditis, especially if recurrent, autoimmune or hypersensitivity states are entailed [30]. This association may be of specific interest to CD, where a pathophysiological link with immune disturbances is often recognized. In most cases of idiopathic pericarditis, recovery is complete, but fatal cases and constrictive pericarditis have been reported [28,31]. Some early reports suggested that the recurrence of pericarditis was unusual [32]. However, recent studies suggested that recurrence is common, particularly in the first six months following the initial episode [30]. Little is known about the underlying link between CD and pericarditis.

Faizallah et al. reported three cases of acute recurrent pericarditis as a presenting manifestation of CD. In the three patients presenting with acute recurrent pericarditis of unknown cause, the investigation revealed evidence of malabsorption due to CD [1]. Two patients responded to a GFD and corticosteroid therapy, while the third responded to a GFD alone. The CD was diagnosed based on histological subtotal villous atrophy and clinical and biochemical responses to a GFD, associated with HLA-B8 in all and DRW3 in two cases [1].

Dawes et al. reported two cases of recurrent pericarditis as the presenting feature of CD. Treatment with a GFD resolved gastrointestinal symptoms and signs of pericarditis. The authors concluded that jejunal biopsy should be considered in patients with recurrent pericarditis of an unknown cause [33].

Laine et al. reported a case of clear-cut diagnoses of CD and recurrent idiopathic pericarditis. The 63-year-old male patient had no recurrence of pericarditis three months after the beginning of a GFD. The authors supported the idea that recurrent pericarditis may be an immune manifestation of CD [34]. This finding may be significant as it suggests that pericarditis could be a symptom of CD, which could have implications for the diagnosis and treatment of both conditions.

Finally, Walker et al. reported immunologic abnormalities in CD, including pericarditis, which may be secondary manifestations of the disease itself [4].

Meanwhile, Elfstrom et al. investigated the risk of myocarditis, cardiomyopathy, and pericarditis in patients with CD from a general population cohort. Through the Swedish national registers, they identified 9363 children and 4969 adults with a diagnosis of CD (1964–2003). They found no association between CD, later myocarditis, cardiomyopathy or pericarditis. However, they restricted their analyses to heart disease diagnosed in adult hospitalized patients, particularly in departments of cardiology and medicine. Secondly, prior cardiomyopathy of any type and pericarditis were associated with later CD, meaning that they could be presenting manifestations of CD that was diagnosed later [8].

### 4.5. Celiac Disease and Myocarditis

Inflammatory cardiomyopathies, including pericarditis (especially if recurrent) and myocarditis, can occur in CD, representing a rare complication of this disease [1,2,3,4,15].

CD is usually diagnosed in children exhibiting typical gastrointestinal manifestations [17]. However, extraintestinal and multisystemic involvement can occur, with neurological, psychiatric, dermatologic, ocular, musculoskeletal, endocrine, pulmonary, and reproductive alterations [15]. Moreover, a damaged CV system can be observed in untreated CD patients. The risk of irreversible heart damage in unrecognized patients with CD increases with age and with the duration of gluten exposure [9].

The combination of CD and inflammatory cardiomyopathies is unlikely to be due to chance, since both are uncommon [35].

Interestingly, the majority of CD patients with myocarditis and/or recurrent pericarditis appear to respond to dietary therapy. In these cases, a swift diagnosis and implementation of a GFD and/or immunosuppressive therapy (in refractory cases) is necessary to avoid progression into severe CV complications like dilated cardiomyopathy, heart failure, and malignant cardiac arrhythmias.

Therefore, CD might have a direct pathogenetic effect on developing pericarditis and/or myocarditis [2,4,10,13,14,35,36,37,38,39].

As for other autoimmune disorders associated with CD, the most likely hypothesis is an autoimmune cross-reaction simultaneously involving the pericardium/myocardium and the small intestine. Specifically, the immunological basis of CD is secondary to the formation of immune complexes because of intestinal mucosal damage, with tissue transglutaminase being a potential antigenic target. Circulating immune complexes, which correlate with intestinal damage severity and the associated systemic manifestations, could be responsible for developing pericarditis and myocarditis [12,15,40]. Immune complex deposition or an antigen crossing the damaged intestinal mucosa and stimulating an immune response cross-reactively with a host pericardial or myocardial antigen could explain this link. Additionally, pericarditis and myocarditis can arise against a favorable genetic background, such as that known for CD (HLA-DQ2/8), potentially in association with any further unexplored genetic polymorphisms that could elicit latent inflammatory cardiomyopathies. Other reported hypotheses include the following: (i) chronic malabsorption resulting in micronutrient deficiencies (i.e., thiamine, calcium, selenium, and carnitine), which are essential components of myocardial contractility and associated gut edema due to cardiac failure; (ii) myocardial damage caused by infectious agents that pass through the malfunctioning intestinal barrier; (iii) immunodepression secondary to the common immunoglobulin and intestinal lymphocytes deficiency due to malabsorption; and (iv) chronic anemia leading to a hyperdynamic state due to neurohormonal activation secondary to peripheral vasodilatation and progressing to cardiac failure [2,10,12,13,35,37].

Understanding the link between CD and pericarditis and/or myocarditis has meaningful diagnostic and therapeutic implications.

Indeed, early diagnosis with screening tests can prevent severe complications and is essential to preventing disease progression. It has been demonstrated that strict adherence to a GFD can reverse cardiac changes and improve quality of life.

Therefore, patients who present with myocarditis and/or pericarditis (especially if recurrent) for unexplained reasons should be thoroughly investigated. Specifically, an accurate family and personal history, proper analysis of associated signs and symptoms (i.e., abdominal pain, bloating, diarrhea, steatorrhea, weight loss, fatigue, iron deficiency, anemia, and extraintestinal symptoms including dermatitis herpetiformis, osteoporosis, infertility, and neurological symptoms), serological tests, and/or duodenal-jejunal biopsy should be considered in patients with myocarditis and/or pericarditis of an unknown etiology in order to exclude CD [2,30,31,32].

On the other hand, when suspecting pericarditis in CD patients, the presence of pericardial chest pain and pericardial rubs should be investigated. An electrocardiogram should be performed to detect signs such as PR depression and new widespread ST elevation. Moreover, transthoracic echocardiography should be performed to detect pericardial effusion (new or worsening).

Regarding the diagnosis of myocarditis in CD patients with a suggestive clinical presentation (i.e., chest pain, heart failure symptoms and signs, arrhythmias), an electrocardiogram, a transthoracic echocardiogram, and a CMR should be performed. In a few selected cases (i.e., unexplained acute heart failure with hemodynamic compromise or life-threatening ventricular arrhythmias/conduction disorders of an unknown etiology), there may also be a need for endomyocardial biopsy [11,20,21].

Based on the concepts above, a multidisciplinary approach involving cardiologists and gastroenterologists in this area is required to diagnose and treat these diseases promptly.

Further research is needed to explore the underlying pathogenic mechanisms that connect these CV conditions and CD, as this association has been underreported in the literature.

## 5. The Inflammatory Effect of SGLT2 Inhibitors in Autoimmune Diseases

SGLT-2 inhibitors, which are primarily known for their glucose-lowering properties, also show considerable potential as anti-inflammatory agents, which could be important in the management of autoimmune diseases [41,42]. Previous studies suggest that SGLT-2 inhibitors reduce the levels of circulating inflammatory factors, decrease inflammation within the arterial wall, inhibit foam cell formation, limit macrophage infiltration, and promote plaque stability—mechanisms that collectively impede the development and progression of atherosclerosis [42]. Although in vitro studies have reduced inflammatory responses in immune cell models, in vivo SGLT2 inhibitors have demonstrated further anti-inflammatory activity by reducing MPO activity in experimental models [42]. These findings highlight the potential of SGLT-2 inhibitors as adjunctive anti-inflammatory therapies and suggest broader applications in the management of autoimmune and inflammatory cardiomyopathies.

## 6. Dietary Recommendations

Case reports and the limited observational studies available in the current literature clearly support the adoption of a strict gluten-free diet [17,18]. There is a theoretical basis for the potential use of dietary supplements and even probiotics based on the pathophysiological mechanisms discussed above [17,18]. Further studies are needed to assess the efficacy and safety of these supplements in preventing relapses.

## 7. Clinical Implications

Recurrent episodes of myocarditis and pericarditis may represent an extracardiac manifestation of celiac disease. Although the evidence is not particularly strong, screening for celiac disease should be considered as part of the diagnostic workup for inflammatory conditions such as pericarditis and myocarditis. A strict gluten-free diet could prevent the recurrence of these events.

## 8. Limitations

This review has several limitations. Firstly, this review mainly included a small number of case reports and case series and only one observational study. Secondly, the sample size is very limited, which limits the possibility of establishing a solid cause–effect relationship between CD and inflammatory CV diseases. Thirdly, in most cases, the diagnosis of myocarditis was based on clinical and echocardiographic assessment, without the involvement of CMR imaging. Finally, patients with heart failure were not treated with optimized medical therapy according to the latest evidence-based guidelines [37].

## 9. Conclusions

Myocarditis and/or pericarditis, especially if recurrent, may complicate CD. The most likely hypothesis is an autoimmune reaction involving both the pericardium/myocardium and the small intestine. Nevertheless, other unexplored mechanisms cannot be excluded. Understanding the underlying link connecting CD and pericarditis and/or myocarditis has essential diagnostic and therapeutic implications. Patients who present with myocarditis and/or pericarditis (especially if recurring) with unknown etiologies should be thoroughly investigated. Indeed, CD patients usually experience resolution of these inflammatory cardiac disorders after starting a strict GFD. Further studies on the clinical impact of dietary supplements and SGLT-2 inhibitors on inflammatory cardiomyopathies associated with celiac disease are needed.

## Figures and Tables

**Table 1:** Case report and observational studies included in this review.

Authors	Year	Study Design	Sample Size	Celiac-Related Inflammatory Cardiomyopathy	Cardiac Diagnosis	Diagnosis of Celiac Disease	Therapy	Resolution
Faizzalah et al. [1]	1982	Case series	3	Recurrent pericarditis	ECG, TTE	Jejunal Biopsy	GFD and steroids	yes
Dawes et al. [8]	1981	Case series	2	Recurrent pericarditis	ECG, TTE	Jejunal biopsy	GFD	Yes
Laine et al. [9]	1984	Case report	1	Recurrent pericarditis	ECG, TTE	Jejunal biopsy	GFD	Yes
Mannarino [10]	2022	Case report	1	Myocarditis with AV block	ECG, TTE	Antibodies and jejunal biopsy	GFD	Yes
Frustaci et al. [2]	2002	Observational study	9	Myocarditis resulted in heart failure or cardiac arrhythmias	ECG, TTE, Ventriculography, Endomyocardial biopsy	Antibodies and jejunal biopsy	GFD with or without immunosuppressive therapy	Yes
Patel et al. [11]	2018	Case report	1	Myocarditis resulted in heart failure	ECG, TTE, CMR, endomyocardial biopsy	Antibodies and jejunal biopsy	GFD, supportive therapy, prophylactic measures for heart failure	No
Milisavljević et al. [10]	2012	Case report	1	Myocarditis resulted in heart failure	ECG, TTE	Antibodies and jejunal biopsy	GFD, supportive therapy	No
Poddar et al. [12]	2014	Case report	2	Myocarditis resulted in heart failure	ECG, TTE	Antibodies and jejunal biopsy	GFD, supportive therapy	No
Marcolongo et al. [13]	2021	Case report	1	Myocarditis resulted in heart failure	ECG, TTE, CMR, endomyocardial biopsy	Antibodies	GFD and immunosuppressive therapy	Yes
Mehra et al. [14]	2022	Case report	1	Myocarditis resulted in heart failure	ECG, TTE	Antibodies and jejunal biopsy	GFD and supportive therapy	Yes

**Table 2:** Tool for evaluating the methodological quality of case reports and case series of the current review suggested by Murad et al. [6].

Domains	Leading Explanatory Questions	Cases and Cases Series Included in the Current Review	Score
Selection	1. Does the patient(s) represent(s) the whole experience of the investigator (center) or is the selection method unclear to the extent that other patients with similar presentation may not have been reported?	Yes	1
Ascertainment	2. Was the exposure adequately ascertained?	Yes	1
	3. Was the outcome adequately ascertained?	Yes	1
Causality	4. Were other alternative causes that may explain the observation ruled out?	Yes	1
Causality	5. Was there a challenge/rechallenge phenomenon?	No	0
	6. Was there a Dose–Response effect?	No	0
	7. Was follow-up long enough for outcomes to occur?	Yes	0
Reporting	8. Is the case(s) described with sufficient details to allow other investigators to replicate the research or to allow practitioners make inferences related to their own practice?	Yes	0
